# Onco-anxiology: a proposal for an anxiety-focused, longitudinal framework within cancer care

**DOI:** 10.3389/fpsyg.2026.1939608

**Published:** 2026-09-18

**Authors:** Chirag Jain

**Affiliations:** Mindful Gurukul Private Limited, Mumbai, India

**Keywords:** cancer anxiety, cancer fear, fear of cancer recurrence, heart rate variability, longitudinal anxiety care in oncology, onco-anxiology, psycho-oncology, supportive care

## Abstract

**Background:**

Anxiety is among the most common forms of psychological distress in people with cancer, yet in routine practice it is usually addressed within a broad “distress” construct rather than as a distinct clinical target.

**Objective:**

This Perspective examines whether anxiety warrants a dedicated, longitudinally organised clinical focus, and proposes onco-anxiology as such a framework within psycho-oncology.

**Key points:**

Anxiety in cancer is phenomenologically distinct from depression, frequently oriented toward realistic future threat such as fear of cancer recurrence, follows a patterned course at the population level, and is shaped by cancer-specific mechanisms, including conditioned anticipatory symptoms and the overlap between somatic anxiety items and treatment side effects; existing screening is generally organised around undifferentiated distress rather than anxiety specifically, and unaided clinician recognition and help uptake are limited. Onco-anxiology is defined by three commitments: anxiety-specific targeting, a longitudinal lens, and explicit attention to cancer-specific anxiety mechanisms. Physiological measurement is positioned as a research direction and an optional adjunct rather than a defining feature, and the framework is offered as a way of organising care within psycho-oncology rather than as a claim to a new sub-specialty.

**Conclusion:**

A focused framework may improve identification and treatment of anxiety in oncology. The term is proposed as a testable organising concept, not an established field, and a research agenda is outlined.

## 1 Introduction

Clinically significant anxiety is a frequent experience across the cancer continuum. In the largest interview-based meta-analysis to date, anxiety disorders were present in roughly one in 10 patients, while some combination of mood disorders — depression, anxiety, or adjustment disorder — affected 30%–40% of patients in oncological, haematological, and palliative-care settings (Mitchell et al., 2011). Anxiety *symptoms* short of a formal disorder are more common still. Untreated, anxiety is associated with poorer sleep, greater symptom burden, reduced treatment adherence, and avoidance of appointments and surveillance.

Modern psycho-oncology, founded by Jimmie Holland and colleagues, established that the psychological dimension of cancer is real, measurable, and treatable, and embedded distress screening into routine care (Roth et al., 1998). Building on that foundation, this Perspective asks a narrower question: within that broad field, does anxiety warrant its own focused clinical lens? I argue that four features of anxiety in cancer — its distinctiveness, its patterned trajectory, its assessment challenges, and its cancer-specific mechanisms — together justify testing a focused framework, here termed *onco-anxiology*.

## 2 Anxiety is distinct from depression — and cancer alters its form

Anxiety and depression are frequently grouped as “distress,” yet they differ phenomenologically and therapeutically. Anxiety is characterised by hypervigilance, anticipatory dread, and autonomic arousal; depression by low mood, anhedonia, and withdrawal. Screening and treatment pathways optimised for depression — the more visible and more studied condition — do not automatically serve anxiety. Unaided clinician recognition of psychological morbidity is limited (Passik et al., 1998), and internally experienced hypervigilance is inherently less observable than overt low mood, which may contribute to under-detection of anxiety specifically.

This contrast, however, is drawn from general psychopathology and is not by itself specific to oncology. If it were the whole argument, the appropriate response would be better delivery of existing anxiety-focused care within psycho-oncology rather than a named sub-domain. The stronger claim made here is that the medical context of cancer alters the aetiology, the measurement, and the management of anxiety in three ways that have no clean equivalent in primary anxiety disorders.

First, some anxiety in cancer is iatrogenically conditioned. Repeated pairing of clinic cues — the smell of the infusion suite, the sight of the cannula — with cytotoxic treatment produces anticipatory nausea and vomiting in roughly one in four patients receiving chemotherapy. This is a classically conditioned response rather than a pharmacological one: it responds poorly to antiemetics, and consensus guidance recommends behavioural techniques and benzodiazepines instead, with optimal control of acute emesis as the principal preventive strategy (Dupuis et al., 2017). The conditioned stimulus is created by treatment, the response is somatic, and the management pathway sits with the oncology team rather than with mental-health services. No equivalent mechanism operates in generalised anxiety disorder.

Second, anxiety in cancer is measured against a noisy somatic background. The physical items on generic anxiety instruments — palpitations, breathlessness, restlessness, disturbed sleep, gastrointestinal upset — are also produced by the disease and its treatment: pain, anaemia, deconditioning, opioids, antiemetics and corticosteroids. Corticosteroids, given routinely with many regimens, can themselves precipitate agitation, akathisia, insomnia and frank psychiatric disturbance in oncology patients (Seymour et al., 2025). The same questionnaire score may therefore reflect psychological threat, drug effect, or disease burden, and separating them requires oncological knowledge that generic anxiety pathways do not assume. Psychosocial distress in this population is accordingly described as multidimensional and inseparable from physical symptom burden (Shivappa et al., 2024).

Third, anxiety and physical symptoms are bidirectionally coupled. Anxiety at the time of treatment increases the likelihood and severity of nausea, and poorly controlled nausea and pain in turn intensify anticipatory anxiety before subsequent cycles. Treating the anxiety and treating the symptom are therefore not separable tasks: effective anxiolysis may depend on antiemetic and analgesic optimisation, and adequate symptom control may depend on addressing anxiety. Taken together, these features describe anxiety that is partly created by treatment, partly obscured by it, and partly treatable through it. That combination — rather than the hypervigilance–anhedonia contrast alone — is what distinguishes onco-anxiology from anxiety care that merely happens to be delivered in an oncology setting.

## 3 A realistic fear, oriented to the future

A defining feature of much cancer-related anxiety is that it concerns a genuinely possible future event rather than a cognitive distortion. Fear of cancer recurrence (FCR) is among the most commonly reported unmet needs in survivorship; across studies, any level of FCR has been reported in a wide range (approximately 39%–97%), with moderate-to-high levels in a substantial subset, and it tends to persist over the survivorship trajectory (Simard et al., 2013). Validated measurement, such as the Fear of Cancer Recurrence Inventory, has enabled more precise characterisation (Simard and Savard, 2009). Because the feared event is real, the therapeutic task is less about correcting overestimated threat than about building the capacity to live alongside uncertainty — an emphasis operationalised and tested in interventions such as ConquerFear, a theoretically based psychosocial treatment evaluated in a randomised trial (Butow et al., 2017). Anxiety-focused framing keeps this distinction central.

## 4 A patterned trajectory of vulnerability

Anxiety is not static across the disease course; its character shifts in clinically recognisable ways. Pre-diagnostic anxiety centres on uncertainty about whether a threat exists at all. Anxiety at diagnosis is acute. Treatment-phase anxiety is procedural and anticipatory. A counterintuitive rise is frequently described at treatment completion, when the protective structure of frequent clinical contact withdraws. In survivorship, FCR can persist for years and often intensifies around surveillance imaging. At recurrence, anxiety may equal or exceed that at initial diagnosis. These are best described as population-level points of increased vulnerability rather than as a predictable individual course. Between-patient heterogeneity is substantial: the shape of any one patient’s anxiety depends on prognosis, treatment modality, age, social support, psychiatric history, symptom burden, perceived risk and cancer type, and some patients will show none of the pattern described above. What the trajectory supports is the timing of assessment, not the prediction of an individual’s experience. A framework that follows anxiety longitudinally can anticipate need at these points rather than react to crisis.

## 5 Assessment: distress screening versus anxiety-specific screening

Routine psychosocial screening in oncology is typically organised around undifferentiated distress — for example, the single-item Distress Thermometer, developed for rapid identification in busy clinics and widely adopted (Roth et al., 1998). Such tools are valuable but are not designed to characterise anxiety specifically or to track its evolution across the trajectory. Moreover, identification does not guarantee treatment: a substantial proportion of patients who screen positive for distress decline offered help, for reasons including perceived burden and stigma (Clover et al., 2015). An anxiety-specific, longitudinally structured approach would need to demonstrate added value over existing distress screening while addressing these uptake barriers. Two qualifications matter here. The distress construct was developed as a broad, pragmatic indicator for rapid case-finding, not as a substitute for construct-specific assessment; showing that a distress screen does not characterise anxiety is therefore not a criticism of the distress model but a description of the division of labour between screening and subsequent assessment. The gap identified here lies downstream of the screen: what follows a positive result is frequently a general referral rather than an anxiety-specific assessment, and the screen is rarely repeated on a schedule tied to the disease trajectory. The argument is therefore for what should be built on top of distress screening, not for replacing it.

## 6 A physiological dimension: a research direction

Unlike some psychological constructs, anxious arousal has objective physiological correlates — autonomic activation, hypothalamic–pituitary–adrenal axis engagement, and changes in heart rate variability. These offer, in principle, opportunities for objective assessment and for interventions that respond to a patient’s real-time physiological state. This is presented as a direction for development rather than established practice: the evidence base for physiologically grounded and adaptive interventions in oncology is early, and claims of clinical readiness would require rigorous, adequately powered evaluation.

Oncology-specific evidence, while limited, is no longer absent. In breast cancer, patients with lower vagally mediated heart rate variability report significantly higher anxiety than those with higher variability, alongside a shifted inflammatory profile, supporting HRV as a candidate correlate of anxious arousal in this population and not only in psychiatric samples (Zhang et al., 2025). Interventionally, a randomised wait-list-controlled pilot in cancer survivors found that HRV biofeedback training improved HRV coherence and reduced cancer-related symptom burden, including stress and distress (Burch et al., 2020). A subsequent three-arm randomised controlled trial of a four-week remote, smartphone-delivered HRV biofeedback protocol in 60 women with primary breast cancer reported increased low-frequency HRV, improvement in sleep quality sustained at 6 months, and reduced anxiety relative to both active and waitlist controls (Dolgilevica et al., 2025). Continuous monitoring also appears tolerable in sicker populations: in an outpatient palliative-care sample of patients with advanced cancer and their family caregivers, consumer-grade wearables used an HRV-derived stress algorithm to trigger brief momentary symptom surveys, achieving 73% daytime wear adherence and positive participant appraisal (Schuler et al., 2023).

These studies establish feasibility and a preliminary intervention signal; they do not establish diagnostic validity. To my knowledge no published oncology study has validated an HRV-derived index against a reference-standard anxiety assessment, defined clinical thresholds, or shown that physiological data alter clinical decisions. The closest work applies machine learning to wrist-worn wearable signals in oncology patients, predicting anxiety among nine self-rated symptoms with F1 scores above 70% in a small sample (Moscato et al., 2025); the authors describe the findings as preliminary, and the comparator is a self-rated symptom score rather than a reference-standard anxiety assessment. Association between reduced HRV and anxiety is well established in psychiatric populations; what is absent in oncology is a validated index with defined thresholds. Samples are small, predominantly female, predominantly breast cancer, and drawn largely from survivorship rather than active treatment. The signal is also non-specific and heavily confounded in this population: anthracycline and trastuzumab cardiotoxicity, anaemia, deconditioning, autonomic neuropathy, beta-blockade, opioids and corticosteroids all move HRV independently of anxiety, and HRV is reduced by advanced disease itself. Physiological measurement is therefore advanced as a research direction and an optional adjunct to self-report — a hypothesis about what may become measurable — rather than a claim of present clinical readiness. For the same reason, it is deliberately not treated below as a defining commitment of the framework. A framework should not rest a third of its definition on evidence its own author describes as preliminary. The proposal is therefore constructed so that it stands or falls on clinical logic alone and would remain intact if physiological indices proved uninformative; the physiological question is carried instead by the research agenda (H3, H4).

## 7 A proposed framework: onco-anxiology

Drawing these threads together, I propose *onco-anxiology* as a focused framework organised around three commitments. *Specificity*: anxiety is treated as a distinct clinical target, differentiated from depression, adjustment disorder, and undifferentiated distress. *A longitudinal lens*: anxiety is assessed and managed as an evolving phenomenon across the full cancer timeline, with attention to population-level points of increased vulnerability. *Mechanism specificity*: the cancer-specific mechanisms set out in Section 2 — conditioned anticipatory symptoms, somatic confounding of anxiety measurement, and anxiety–symptom coupling — are treated as explicit clinical targets rather than as background to general symptom management. Physiological measurement, where feasible, is an optional adjunct and a research priority, not a defining pillar and not a requirement for practising the framework. Figure 1 sets out how these commitments map onto the cancer trajectory.

**Figure 1:**
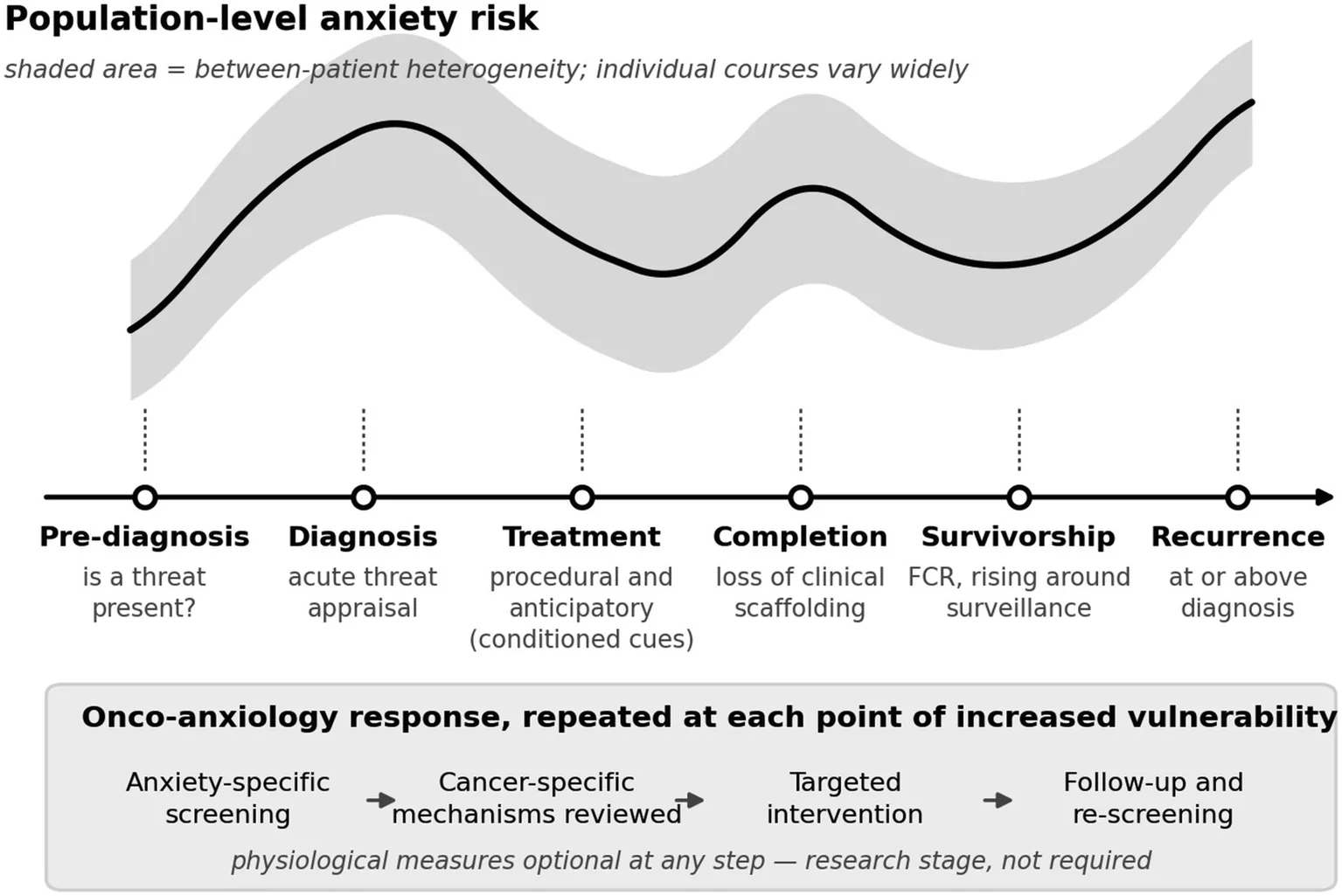
The onco-anxiology framework applied across the cancer trajectory. The upper trace indicates how anxiety risk is expected to vary at the population level, with the shaded band representing substantial between-patient heterogeneity; individual courses may depart from it entirely. The lower panel shows the clinical response repeated at each point of increased vulnerability: anxiety-specific screening, review of cancer-specific mechanisms, targeted intervention, and follow-up, with physiological measures optional at any step. The curve is a hypothesis to be tested prospectively (H2), not an established finding.

## 8 Relationship to psycho-oncology, and what would justify a focused framework

Onco-anxiology is proposed as a focused framework within psycho-oncology, not a competing discipline. Psycho-oncology is deliberately comprehensive, addressing depression, anxiety, adjustment, delirium, existential distress, and family and caregiver impact. The proposed framework differs in three respects: it narrows the target to anxiety, structures assessment around the whole timeline rather than around the point of referral, and treats cancer-specific anxiety mechanisms as explicit clinical objects rather than as background. The framework earns its name only if it proves empirically useful — that is, if organising care around this lens improves identification and outcomes relative to usual psycho-oncology practice. Table 1 summarises the difference in emphasis.

**Table 1:** Psycho-oncology and the proposed onco-anxiology framework compared.

Dimension	Psycho-oncology	Onco-anxiology (Proposed)
Clinical Target	Broad psychological care: depression, anxiety, adjustment, delirium, existential distress, family and caregiver impact	Anxiety specifically, differentiated from depression, adjustment disorder, and undifferentiated distress
Screening	Undifferentiated distress screening (e.g., the single-item Distress Thermometer)	Anxiety-focused screening, including fear of cancer recurrence (FCR)
Assessment Cadence	Episodic, often triggered by a single point of contact or by referral	Longitudinal, anchored to population-level points of increased vulnerability across the cancer timeline
Measurement	Self-report questionnaires and clinical interviews	Questionnaires and clinical interview, with physiological measures as an optional, research-stage adjunct
Cancer-Specific Mechanisms	Addressed within general symptom management	Explicit targets: conditioned anticipatory symptoms, somatic confounding of anxiety items, anxiety–symptom coupling

The difference lies in target, time frame, and which mechanisms are treated as explicit clinical objects. Onco-anxiology is a narrower organisation of care within Psycho-oncology, not a separate discipline.

Two objections follow, and both deserve direct answers. The first is that this describes a reorganisation of knowledge already held within psycho-oncology rather than a new construct. The second is that the same reasoning would license an indefinite series of sub-domains — for depression, trauma, grief or stress — with terminological fragmentation as the result.

On the first: I accept it, and it is not fatal. A framework is a way of organising assessment and care, and reorganisation is what it offers. Nothing here claims that the phenomena described are unknown to psycho-oncology; the claim is that they are not currently organised in a way that produces anxiety-specific, trajectory-anchored action, and that organising them so generates testable predictions. Psycho-oncology itself began as a focused framing of psychological care within a broader clinical field, and its value was established by what it produced rather than by prior agreement that a new framing was warranted.

On the second: the objection has force only in the absence of criteria, so criteria are offered in Table 2. A phenomenon merits focused organisation if it is generated or shaped by mechanisms specific to cancer and its treatment, if existing instruments systematically mis-measure it in this population, if its management requires actions outside standard psychological care, and if its risk is patterned by the disease trajectory rather than distributed evenly. Anxiety in cancer meets all four.

**Table 2:** Proposed criteria for treating a psychological phenomenon in cancer as a focused framework, applied to anxiety.

Criterion	What it requires	Anxiety in cancer
Distinct mechanism	The phenomenon is generated or shaped by processes specific to cancer or its treatment	Conditioned anticipatory symptoms; treatment- and corticosteroid-induced arousal (Section 2)
Distinct measurement problem	Existing instruments systematically mis-measure it in this population	Somatic anxiety items confounded by disease, pain, anaemia and drug effects (Section 2)
Distinct treatment pathway	Effective management requires actions outside standard psychological care	Antiemetic, analgesic and steroid-regimen decisions taken by the oncology team
Distinct temporal structure	Risk is patterned by the disease trajectory rather than distributed evenly	Elevations at diagnosis, treatment completion and surveillance (Section 4)

The criteria are offered so that the proposal can be contested on stated grounds; other phenomena may also satisfy them.

I accept openly that other phenomena may also meet them — treatment-related traumatic stress is a plausible candidate — and that this would be a reasonable outcome rather than a failure of the criteria, provided each such framework generated predictions capable of failing. The risk worth guarding against is not the number of named frameworks but the naming of frameworks that cannot be falsified. A label that carries its own discontinuation conditions, as set out in Section 10, is a scientific object rather than a claim to territory.

What is claimed here is correspondingly modest. Onco-anxiology names a framework, not a sub-specialty: it implies no separate services, no separate practitioners, no separate training pathway, and no separation from psycho-oncology, within which it sits entirely. Readers who prefer a plainer designation — anxiety-focused psycho-oncology, or longitudinal anxiety care in oncology — lose nothing of the substance; the term is offered as compact shorthand for the three commitments above, and should be judged, and if necessary discarded, on whether it proves useful.

## 9 Implementation and equity

A framework that presumed continuous physiological monitoring would risk being adoptable only where it is least needed, so its implementation demands should be stated plainly. Four barriers dominate in low-resource oncology. *Devices*: validated hardware, charging, data storage and technical support carry recurring costs, and in many settings patients pay out of pocket. *Personnel*: psycho-oncology staffing is thin — in an Indian tertiary centre with routine distress screening, 53.5% of patients screened at moderate-to-extreme distress yet only 8.6% were referred to psycho-oncology services, with providers citing time constraints, generic screening tools, patient reluctance about mental-health referral, and reliance on personal clinical judgement (Normen et al., 2021). *Clinic time*: consultations are short and volumes high, so any instrument added to the visit competes directly with throughput. *Continuity*: a longitudinal lens assumes patients return to the same centre, which is often untrue where travel distance and cost drive attrition and treatment abandonment.

All three defining commitments — anxiety-specific targeting, a longitudinal lens, and attention to cancer-specific mechanisms — can be practised without any technology, and cost almost nothing to implement. Only the optional physiological adjunct carries a hardware cost, which is a further reason not to treat it as constitutive. A tiered model follows. *Tier 1 (no technology)*: a brief anxiety-specific screen in the local language, administered by a nurse or trained non-specialist counsellor at four fixed trajectory anchors — diagnosis, mid-treatment, treatment completion, and first surveillance visit — each with a defined action for a positive result, since detection without an actionable pathway simply reproduces the referral gap described above. *Tier 2 (shared hardware)*: short seated HRV recordings on a single clinic-owned device during infusion or waiting time, converting a per-patient capital cost into a per-clinic one. *Tier 3 (continuous monitoring)*: wearables, restricted for now to research settings and services able to support them.

Physiologically informed intervention may in fact scale more readily than physiological measurement: the paced-breathing protocol tested by Dolgilevica et al. (2025) was delivered remotely through a smartphone, and protocolised, group-delivered relaxation training can be task-shifted to nursing and counselling staff. Any evaluation of onco-anxiology in such settings should report implementation outcomes — screening completion, referral uptake, added clinician minutes per patient, and retention — alongside symptom outcomes, and should be conducted where cancer incidence is rising fastest rather than only where the enabling technology already exists.

## 10 A research agenda

Priorities include: (1) *construct validation* — whether anxiety-specific, longitudinally structured screening improves detection and treatment relative to standard distress screening; (2) *trajectory characterisation* — prospective mapping of anxiety across the full timeline, including the under-studied pre-diagnostic and treatment-completion periods; (3) *physiological assessment* — whether continuous physiological signals add information beyond self-report; (4) *adaptive intervention* — rigorous randomised evaluation of physiologically adaptive interventions against static comparators, with attention to equity and generalisability; and (5) *implementation* — integration into oncology workflows without increasing clinician burden.

Stated as testable hypotheses, each with the finding that would falsify it:

H1 (detection): In a cluster-randomised comparison of anxiety-specific longitudinal screening against the single-item Distress Thermometer, the anxiety-specific arm will identify a higher proportion of patients with clinically significant anxiety and shorten median time from positive screen to first intervention at 6 months. *Falsified if* detection rates and time-to-intervention do not differ.

H2 (trajectory): At the population level, in a prospective cohort measured monthly from diagnosis to 24 months, anxiety will follow a non-monotonic course with a secondary peak within 3 months of treatment completion and transient elevations in the fortnight preceding surveillance imaging, independent of concurrent depression scores. *Falsified if* the trajectory is flat or simply tracks depression.

H3 (incremental information): Resting vagally mediated HRV will add predictive value beyond self-report for fear of cancer recurrence at surveillance, giving a meaningful gain in explained variance after adjustment for age, body-mass index, anaemia, exposure to cardiotoxic therapy, beta-blockade and disease stage. *Falsified if* the increment is null once these confounders are modelled.

H4 (adaptive intervention): A physiologically triggered, just-in-time anxiolytic prompt will outperform the same content delivered on a fixed schedule at 8 weeks; a device-free version delivered at fixed trajectory anchors will be non-inferior to the fixed-schedule digital arm. *Falsified if* triggering confers no advantage over scheduling.

H5 (implementation): In a hybrid effectiveness–implementation evaluation at a high-volume, low-resource centre, nurse-delivered anxiety-specific screening will raise referral uptake above the approximately 9% benchmark reported by Normen et al. (2021) while adding under 2 minutes of clinician time per patient. *Falsified if* uptake is unchanged or staff judge the workflow burden unacceptable.

The construct should be abandoned if anxiety-specific, longitudinally structured screening confers no detection or outcome advantage over undifferentiated distress screening and physiological measures add no information beyond self-report. Retaining a distinct named framework is justified only if it generates predictions of this kind.

Figure 2 places these hypotheses in a staged programme, in which each stage is capable of returning the construct for revision rather than only advancing it.

**Figure 2:**
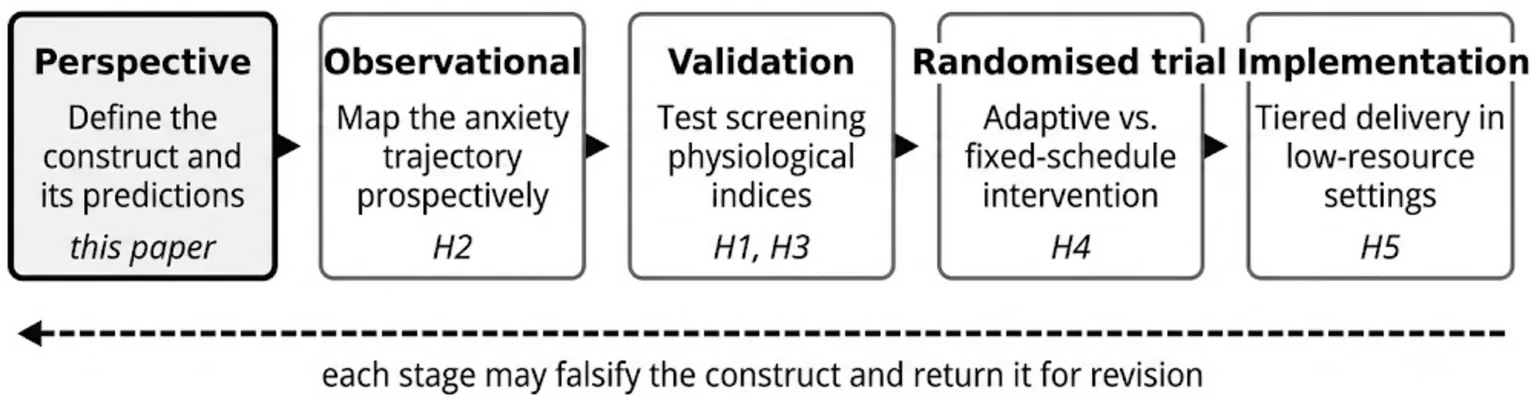
A staged research programme for onco-anxiology, with the hypotheses tested at each stage. Progression is conditional: A negative result at any stage should revise or retire the construct rather than defer it to the next stage.

## 11 Limitations

The proposed framework is conceptual and untested: its central claims are hypotheses requiring evaluation. There is a definitional risk of over-medicalising normal, adaptive fear, and the framework must distinguish impairing anxiety from proportionate concern. Much cited evidence derives from breast-cancer and high-resource settings; generalisability to diverse Indian and low- and middle-income contexts requires direct study. The oncology-specific physiological evidence cited here is likewise drawn from small and restricted samples, predominantly women with breast cancer and mostly in survivorship, and none of it validates a physiological index against a reference-standard anxiety assessment. Physiological measurement should therefore be understood as an optional research direction rather than a defining commitment of the framework. A further risk is that emphasising physiological measurement at all could imply that objective signals are a superior form of evidence to the patient’s own account. They are not. Anxiety in cancer is constituted by existential, prognostic, familial, social, economic, spiritual and relational dimensions that no autonomic index can represent, and physiological data are proposed here only as a supplement to subjective report, never as an arbiter of it. The framework also remains a reorganisation of largely existing knowledge; its justification rests on whether that reorganisation produces better detection and outcomes, which is an empirical question rather than a conceptual one.

## 12 Conclusion

Anxiety in cancer is common, distinct from depression, frequently oriented to realistic future threat, patterned in its population-level trajectory, and shaped by mechanisms specific to cancer and its treatment — yet it is rarely treated as a clinical target in its own right. Onco-anxiology is proposed as a focused, longitudinal, mechanism-aware lens within psycho-oncology to address this gap — a way of organising care rather than a claim to a new sub-specialty, and a testable concept to be judged by evidence rather than assertion. If validated through prospective research, it could provide a structured basis for integrating anxiety-specific assessment and management throughout the cancer care continuum — including in the high-volume, low-resource settings where most patients with cancer are treated.

## Data Availability

The original contributions presented in the study are included in the article/supplementary material, further inquiries can be directed to the corresponding author.
